# Commentary: Radiomics in oncology: A 10-year bibliometric analysis

**DOI:** 10.3389/fonc.2022.891056

**Published:** 2022-07-22

**Authors:** Guoxin Fan, Jiaqi Qin, Huaqing Liu, Xiang Liao

**Affiliations:** ^1^ Department of Pain Medicine, Huazhong University of Science and Technology Union Shenzhen Hospital, Shenzhen, China; ^2^ Guangdong Key Laboratory for Biomedical Measurements and Ultrasound Imaging, School of Biomedical Engineering, Shenzhen University Health Science Center, Shenzhen, China; ^3^ Department of Spine Surgery, Third Affiliated Hospital, Sun Yatsen University, Guangzhou, China; ^4^ Artificial Intelligence Innovation Center, Research Institute of Tsinghua, Pearl River Delta, Guangzhou, China

**Keywords:** radiomics, bibliometrics, latent dirichlet allocation, machine learning, text mining

## Introduction

Indeed, publications concerning radiomic technique for oncology keep increasing over the past decade. In their valuable article, Ding et al. (1) conducted a bibliometric analysis on the field of radiomics in oncology, with the aim of informing beginners and encouraging more researchers to participate in practicing radiomics. Thus, they quantified the contributions of different countries, authors, institutions, and journals to this field, and tried to identify areas of focus and future trends. We congratulated their works, since this was the first bibliometric study of this field and provided the macroscopic landscape for researchers. However, we assumed a summary of the past, the present and the future direction of researches in this filed might also be valuable. Thus, we suggested a machine learning based text classification to identify research topics and demonstrate how they evolved.

## Comment on the findings and discussion

In the study (1), they used CiteSpace to perform keyword analysis, which might help identify the areas of focus and research trend. They found “artificial intelligence,” “tumors,” “classification,” “segmentation,” and “diagnosis” as areas of focus (without “radiomics”), as these terms were mentioned most frequently. They also found that “test-retest”, “sarcoma”, “statistics”, “intensity-modulated radiotherapy”, and “genomics” could be research trends, as these terms had strongest recent citation bursts. Then, they endeavored to interpret how these keywords correlated to radiomics in the discussion section. However, this kind of keyword analysis from CiteSpace was weak at interpreting how these frequently mentioned terms connected each other, as well as clustering all the publications into research topics.

**Figure 1:**
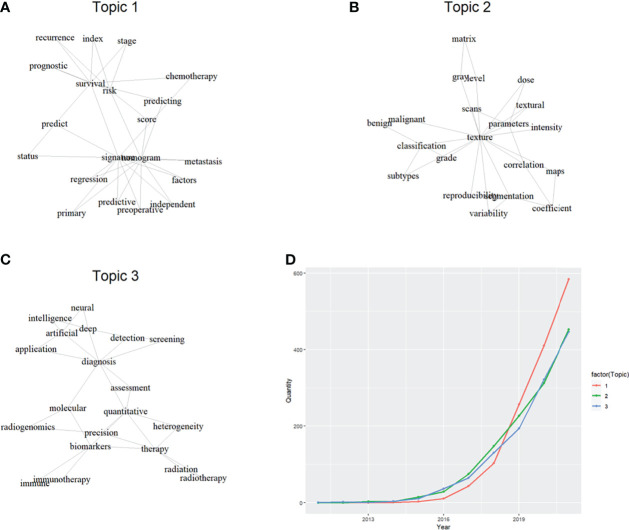
Topic classification by latent Dirichlet allocation and their productivity growth. **(A)**: keyword network of topic 1; **(B)**: keyword network of topic 2; **(C)**: keyword network of topic 3; **(D)**: productivity growth of different topics over years.

Natural language processing (NLP), a popular research field where human language is decoded by machine learning, has been deployed to analyze medical information recently (2). Latent Dirichlet allocation (LDA) is a classic machine learning algorithm of NLP, and has been adopted to obtain research topics based on publications concerning a medical field (3, 4). LDA can build a vocabulary of characteristic terms and then sort publications into different topics (5), which are initially networks of words and needed to be named manually. Thus, we conducted LDA analysis based on 3871 publications (23 publications with NA values were removed) extracted by the search formula [2] in their study (1) into the Web of Science Core Collection (WoSCC) database. We found three major topics in this field, and named them as “utility”, “standardization” and “miscellaneous” (**Figure 1**). The proportion of these three topics were 36.31%, 32.48% and 31.20% respectively. Interestingly, we also found how the productivity of these topics evolved over time.

It seemed that one third of publications were concerned about the reliability of radiomics (Topic 2: standardization), and it remained as the most productive topic between 2016 and 2019. This might not be a surprise, because every novel technique would undergo a standardization process during the early stage. Researchers focused on the standardization of radiomics, as various procedures like segmentation of region of interest, scan protocol, preprocessing details would compromise the consistency of extracted features and the reproducibility of the findings. After the standardization of radiomics, more and more publications focused on the utility of radiomics (Topic 1: utility), and it remained as the most productive topic after 2019. These publications were simply identifying radiomic signature or building nomogram tools for certain clinical issues, but they surged up and contributed to nearly 40% of this field. It seemed that survival prognostication was the major prediction target of developing nomogram based on radiomic features. Interestingly, it should be noticed that publications concerning artificial intelligence and multi-source also grew up quickly (Topic 3: miscellaneous), and they caught up the productivity of Topic 2 after 2020. As radiomics was basically a technique of feature engineering, it seemed that comparisons with other techniques of like deep learning-based feature extraction were often in applications. Additionally, multi-source data like genomics was frequently combined with radiomic features, which theoretically could realize better predictions like higher precisions.

In our perspective, publications of Topic 3 would become a future growing point, as multi-disciplinary researchers were likely to flock into this field and multi-source data would enable more ambitious and achievable goals. First, image segmentation of the region of interest (ROI), a prior step in radiomic workflows, needs the implementation of deep learning technique to improve the applicability of radiomic techniques (6). Manual segmentations of ROI are subjective and time-consuming, which will compromise the generalization of radiomic techniques. Semantic segmentations with deep learning algorithms enable accurate, fast and even multi-class ROIs for tumor contouring. Multi-class ROIs somewhat create multi-source data and expand the research boundary of this field, like the significance of radiomics on the para-carcinoma tissue. Second, multi-source data also includes multi-modality images and multi-omics data, which creates more exploration possibilities of radiomics. Multi-modality images may enable radiomic model to achieve more accurate predictions (7), but the feasibility and the additive value remain unknown for certain circumstance. While radiomics can predict the molecular features for carcinoma, they can also create multi-omics prediction model by integrating genomics, proteomics data etc (8). Anyway, the implication of modelling with multi-source data including radiomics needs massive investigations. Last but may not least, deep learning modelling poses challenges to radiomic modelling, as the former can achieve end-to-end predictions. However, deep learning modelling lacks interpretability, so the interpretable and end-to-end modelling may also become a future trend in the field of radiomics for oncology.

We selected the searching formula [2] instead of the formula [1] in their study to extract data for our LDA analysis, because the formula [2] they proposed seemed to be more directly pertinent to radiomics. Nevertheless, it was not always easy to create a perfect searching strategy, and we admitted that it was also possible that the formula [2] miss some relevant publications. Additionally, other relevant publications not included by the WoSCC database remained as another limitation of our results.

In conclusion, we appreciated their efforts in informing beginners and encouraging more researchers to participate in practicing radiomics by a bibliometric study. Meanwhile, we believed our LDA analysis also provided more in-depth analysis of radiomics in oncology, which should be beneficial to beginners who were eager to quickly grasp the major topics in this field and how they evolved. We hope the previous study (1) and our LDA analysis would shed the light into the future direction of radiomics in oncology.

## Author contributions

GF: writing and data interpreting; JQ: drafting and data analysis; HL: data extraction and revision; XL: study design and critical comments. All authors contributed to the article and approved the submitted version.

## Funding

Guangdong Basic and Applied Basic Research Foundation (2019A1515111171) and National Natural Science Foundation of China (82102640) were received in support of this work. The funders had no role in study design, data collection, data analysis, interpretation, writing of this report and in the decision to submit the paper for publication.

## Conflict of interest

The authors declare that the research was conducted in the absence of any commercial or financial relationships that could be construed as a potential conflict of interest.

## Publisher’s note

All claims expressed in this article are solely those of the authors and do not necessarily represent those of their affiliated organizations, or those of the publisher, the editors and the reviewers. Any product that may be evaluated in this article, or claim that may be made by its manufacturer, is not guaranteed or endorsed by the publisher.
